# Risk Factors and Prognostic Implications of Tumor‐Related Epilepsy in Diffuse Glioma Patients: A Real‐World Multicenter Study

**DOI:** 10.1002/brb3.70510

**Published:** 2025-05-05

**Authors:** Yao Xiao, Zhuang Nie, Jinsha Huang, Jie Zhao, Chengjun Dong, Yan Zou, Zikai Li, Bingqing Yan, Yue Hu, Fan Yang, Jong Woo Lee, Alexander P. Lin, Steven Tobochnik, Min Zhou, Ziqiao Lei

**Affiliations:** ^1^ Department of Radiology, Union Hospital, Tongji Medical College Huazhong University of Science and Technology Wuhan Hubei China; ^2^ Hubei Provincial Clinical Research Center for Precision Radiology & Interventional Medicine Wuhan China; ^3^ Hubei Key Laboratory of Molecular Imaging Wuhan Hubei China; ^4^ Department of Neurology, Union Hospital, Tongji Medical College Huazhong University of Science and Technology Wuhan Hubei China; ^5^ General Hospital of Yangtze River Shipping Wuhan Brain Hospital Wuhan China; ^6^ Cancer Center, Union Hospital, Tongji Medical College Huazhong University of Science and Technology Wuhan Hubei China; ^7^ Department of Neurology Brigham and Women's Hospital Boston Massachusetts USA; ^8^ Department of Radiology Brigham and Women's Hospital Boston Massachusetts USA

**Keywords:** adult diffuse gliomas, epilepsy, OS, PFS, tumor extension

## Abstract

**Purpose:**

The relevance of tumor‐related epilepsy (TRE) to glioma survival is controversial. This study aimed to assess the risk factors and prognostic impact of TRE in adult patients with diffuse gliomas by integrating clinical, radiological, and molecular data.

**Methods:**

This multicenter retrospective study included 1036 adult patients with diffuse gliomas from local hospitals and the POLA Network. Patients were categorized into three prognostic groups: lower‐grade oligodendroglioma/astrocytoma (OD/AC, II–III, IDH‐MT), not otherwise specified or not elsewhere classified (NOS/NEC, II–III, IDH‐WT), and high‐grade gliomas (HGG, IV). Clinico‐radiological, molecular, and therapeutic factors were analyzed using univariate and multivariate logistic regression, with the Cox proportional hazards model applied to identify independent prognostic factors for progression‐free survival (PFS) and overall survival (OS).

**Results:**

TRE occurred in 44.4% of OD/AC patients, 25.8% of NOS/NEC patients, and 16.5% of HGG patients. Multivariate analysis identified age as the only significant independent correlate of TRE in the OD/AC group (OR = 0.961; *p* = 0.004), while the absence of deep structure involvement was independently associated with TRE in the NOS/NEC and HGG groups. In univariate analysis, the presence of TRE was associated with longer PFS and OS across all groups, particularly in the NOS/NEC group, where patients with TRE had a median PFS of 35.2 months compared to 13.6 months in those without TRE (*p* = 0.02), but was not a significant predictor in multivariate analyses. TRE was the only factor significantly associated with maintaining histological grade at recurrence (HR = 0.094; *p* = 0.005).

**Conclusion:**

TRE was not a strong independent prognostic factor after controlling for clinical and molecular tumor features, suggesting that the prognostic relevance of TRE is likely driven by underlying glioma biology and other associated clinical factors.

## Graphical abstract

This multi‐center retrospective study evaluated the risk factors and prognostic impact of tumor‐related epilepsy (TRE) in 1,036 adult patients with diffuse gliomas. Patients were classified into three prognostic groups: OD/AC (IDH‐MT, lower‐grade gliomas), NOS/NEC (IDH‐WT, unclassified grade II‐III), and HGG (high‐grade gliomas). TRE incidence was highest in OD/AC (44.4%) and lowest in HGG (16.5%). Age was an independent predictor of TRE in OD/AC, while the absence of deep structure involvement was linked to TRE in NOS/NEC and HGG. Univariate analysis showed TRE was associated with longer progression‐free survival (PFS) and overall survival (OS), particularly in NOS/NEC patients (median PFS: 35.2 vs. 13.6 months, P = 0.02). However, TRE was not a significant prognostic factor in multivariate models. These findings suggest TRE's prognostic role is likely influenced by glioma biology and clinical factors rather than being an independent predictor of survival outcomes.

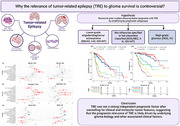

## 1 Introduction

Diffuse gliomas are infiltrative tumors of the central nervous system (Giese et al. 2003), often characterized by tumor‐related epilepsy (TRE) as a common presenting symptom, with a prevalence ranging from approximately 30% to 80% (Maschio and Dinapoli 2012; Rudà et al. 2023). Previous studies have indicated that TRE is more prevalent in glioma patients with tumors located in the frontal and temporal lobes, as these regions are highly associated with epileptogenic activity. Additionally, TRE occurs more frequently in patients with lower histological grades, possibly due to the relatively intact neuronal networks in lower‐grade gliomas, which facilitate epileptiform discharges (Berzero et al. 2021). Moreover, the presence of IDH mutations and cortical infiltration has been linked to an increased likelihood of epilepsy, as metabolic alterations and structural disruption may contribute to neuronal hyperexcitability (Vacher et al. 2023). However, the genetic and molecular factors underlying glioma‐related epileptogenicity remain only partially understood. Additionally, the impact of TRE on glioma prognosis is uncertain (Pallud et al. 2024; Drumm et al. 2023; Guo et al. 2023; Ricklefs et al. 2022; Carstam et al. 2022; Osada et al. 2021; Yuan et al. 2020; Yu et al. 2019; Duan et al. 2018; Neal et al. 2018; Flanigan et al. 2017; Chen et al. 2017; Zhou et al. 2016; Berendsen et al. 2016; Toledo et al. 2015; Wychowski et al. 2013; Bauchet et al. 2010; Lote et al. 1998; Bruno et al. 2024; Chen et al. 2018; Mazzucchi et al. 2022). Some studies have reported an association between preoperative epilepsy and longer survival, while other recent work has demonstrated peritumoral hyperexcitability to promote glioma aggressiveness and to be associated with decreased survival (Mazzucchi et al. 2022; Taylor et al. 2023; Tobochnik et al. 2022; Krishna et al. 2023; Venkatesh et al. 2019). Therefore, it is necessary to improve early epilepsy phenotyping in glioma patients to improve diagnosis and prognosis, surgical decision‐making, optimal use of anti‐seizure medications, and targeted management strategies to maximize quality of life and oncologic outcomes.

With the latest 2021 WHO classification of central nervous system (CNS) tumors, molecular criteria have provided improved definition of adult diffuse gliomas, offering clearer insights into their presentation and prognosis compared to traditional methods based on histologic features alone (Louis et al. 2021). Notably, lower‐grade gliomas and IDH‐mutant (IDH‐MT) gliomas have been consistently associated with better prognosis (Louis et al. 2021). Tumors classified as lower‐grade but without IDH mutations are considered to exhibit heterogeneous tumor characteristics and prognosis. Recently, the cIMPACT‐NOW consortium reached a consensus that IDH‐wild‐type (IDH‐WT) grade II and III astrocytomas exhibiting epidermal growth factor receptor (EGFR) amplification, combined whole chromosome 7 gain and whole chromosome 10 loss (+7/−10), and/or telomerase reverse transcriptase (TERT) promoter mutations should be classified as molecular glioblastomas (GBM) due to their poor survival outcomes (Brat et al. 2018). One report indicated that patients with IDH‐WT grade II astrocytomas meeting the molecular GBM criteria had a median oerall survival (OS) of 42 months, compared to 57 months in patients with IDH‐WT grade II astrocytomas not meeting the molecular GBM definition (Berzero et al. 2021).

Although next generation sequencing is preferred for the integrated pathologic diagnosis of glioma, the necessary resources for this testing remain unavailable in many regions worldwide. As a result, some tumors with less common IDH mutations or molecular GBMs may be incompletely classified. Under the 2021 WHO classification, such gliomas are likely to fall into a category of diffuse glioma not otherwise specified (NOS) or not elsewhere classified (NEC). Considering the strong association between IDH mutations of lower grade gliomas and pre‐operative seizures, we hypothesize that gliomas with NOS/NEC classification have seizure and survival rates in between IDH‐MT glioma and GBM, and that TRE is not independently predictive of prognosis after controlling for prognosis at this group level. The main objective of this study was to evaluate epilepsy risk in stratified prognostic subgroups to reconcile prior studies of TRE and hyperexcitability on survival prognosis. We conduct a real‐world multicenter study aiming to evaluate the clinico‐radiological and molecular characteristics of TRE in patients with diffuse gliomas based on prognostic subgroups: lower‐grade oligodendroglioma/astrocytoma (OD/AC, II–III, IDH‐MT), glioma not otherwise specified or not elsewhere classified (NOS/NEC, II–III, IDH‐WT), and high‐grade gliomas (HGG, IV). The findings help to clarify the prognostic impact of TRE on survival in different glioma cohorts and improve risk stratification for future treatment trials and quality of life studies.

## 2 Methods

### 2.1 Patient Population

This study included patients from two sources. The first group comprised individuals with histologically confirmed gliomas who underwent surgery at Union Hospital, Tongji Medical College, Huazhong University of Science and Technology (Institution 1), and Wuhan Brain Hospital (Institution 2) between January 2013 and December 2021. The inclusion criteria were (1) known preoperative seizure status or clear medical records from which preoperative seizure status could be determined based on well‐documented clinical records or Electroencephalogram (EEG), and (2) available pathology reports allowing the investigation of the histological and genetic features of the gliomas. The exclusion criteria were (1) age under 18 years; (2) WHO grade I gliomas, spinal or infratentorial tumors, and glioneuronal tumors; (3) recurrent gliomas; (4) childhood seizures or seizures attributable to other causes (e.g., head trauma or prior surgery); and (5) stereotactic biopsies and lack of magnetic resonance imaging (MRI) follow‐up at Union Hospital (patients excluded for this criterion were only excluded from prognostic analysis).

The second group consisted of data from the POLA Network (Kamoun et al. 2016), retrieved from ArrayExpress (E‐MTAB‐3892), including glioma patients with epilepsy phenotyping. A total of 124 patients with adult diffuse gliomas according to the 2016 WHO CNS classification were enrolled. Variables included gender, age, seizures at diagnosis, overall survival, and data on histological and molecular markers.

The samples included from Union Hospital and Wuhan Brain Hospital were consecutive sequences, while POLA Network is a public dataset.

### 2.2 Clinico‐Radiological Characteristics

A retrospective evaluation of preoperative seizures, tumor location, midline crossing, and tumor extension status was conducted. Clinical and treatment data were extracted from medical records. All MRI images were presented in random order and independently reviewed by two experienced neuroradiologists (Xiao with 8 years and Zhou with 14 years of brain radiology experience), who were unaware of clinical findings. The final diagnosis was determined by consensus, and inconsistencies between readers A and B were resolved through the decision of another experienced radiologist (Lei with 25 years of brain radiology experience). The midline crossing and tumor extension status were determined based on T2/fluid‐attenuated inversion recovery (FLAIR) imaging. Tumor volume was delineated based on FLAIR and contrast enhanced T1 images in ITK‐SNAP 4.0 software, outlining high signal areas layer by layer while avoiding necrosis and cystic changes. The tumor location was regarded as the lobe in which the majority of the tumor was located. TRE status was then determined using the classification of the International League Against Epilepsy (ILAE 2017) by a neurologist (Jinsha Huang, with over 15 years of experience) (Fisher et al. 2017). Here, TRE was defined as seizures directly attributable to the presence of glioma before surgery. Tumor recurrence was determined according to the RANO criteria, and the period for PFS extended from the date of surgery until the date of recurrence or final follow‐up. OS was determined based on continuous follow‐up calls, with the period recorded from the day of surgery until death or loss to follow‐up.

### 2.3 Molecular Alterations

Samples were collected immediately after surgery and analyzed by the Department of Neuropathology. Isocitrate dehydrogenase 1 (IDH1 R132H) mutations status and the expression levels of P53, Ki‐67, and α‐thalassemia/mental retardation syndrome X‐linked (ATRX) were assessed using Immunohistochemistry staining. For P53 and Ki‐67, the results were divided into two groups: low expression (<10% positive cells) and high expression (≥10% positive cells). pTERT mutations and pMGMT methylation were identified by Sanger sequencing. EGFR gene amplification and 1p/19q codeletion were analyzed by fluorescence in situ hybridization. The molecular profiling was performed as clinically indicated and with available resources at the time of the study. The grade and histological type are determined in accordance with the 2016 WHO classification of CNS tumors and then reclassified by 2021 WHO classification for this analysis.

### 2.4 Extent of Resection

The extent of surgical resection was estimated based on analyses of MRI findings on T2/FLAIR images and contrast enhanced T1 images by a multidisciplinary team within 2 to 3 months after surgery. Gross total resection (GTR) was regarded as the absence of any signal abnormality around the surgical margins; all other conditions were regarded as nongross total resection (non‐GTR).

### 2.5 Statistical Analysis

Baseline patient characteristics were summarized using medians and interquartile ranges (IQRs), as well as frequencies and percentages. Age at surgery was used as a proxy for age at diagnosis. Distributions of characteristics were compared between molecular subgroups by the Mann–Whitney *U* test and independent samples t‐tests for continuous variables, and Fisher's exact test for categorical variables. The relationships of preoperative TRE with other categorical variables were analyzed using univariate and multivariate logistic regression. Kaplan–Meier curves were plotted to evaluate progression‐free survival (PFS) and overall survival (OS). Cox proportional hazards models were used to estimate univariate and multivariate adjusted hazard ratios (HRs) with 95% confidence intervals (CIs), then identify possible predictors of recurrence and death. Proportional risk assumptions were validated by graphical examination and formal testing based on Schoenfeld's residuals. The multivariate model was applied to all patients who had complete information on all tested covariables, that is, no missing data imputation technique was applied. To address the violation of the proportional hazards assumption for the variables, we introduced a time‐dependent covariate by including an interaction term between variables and the logarithm of time in the Cox proportional hazards model. In all analyses, the threshold for statistical significance was *p* < 0.05. Statistical analyses were conducted in R software (R Core Team, 2022).

## 3 . Results

### 3.1 Population Characteristics

**FIGURE 1:**
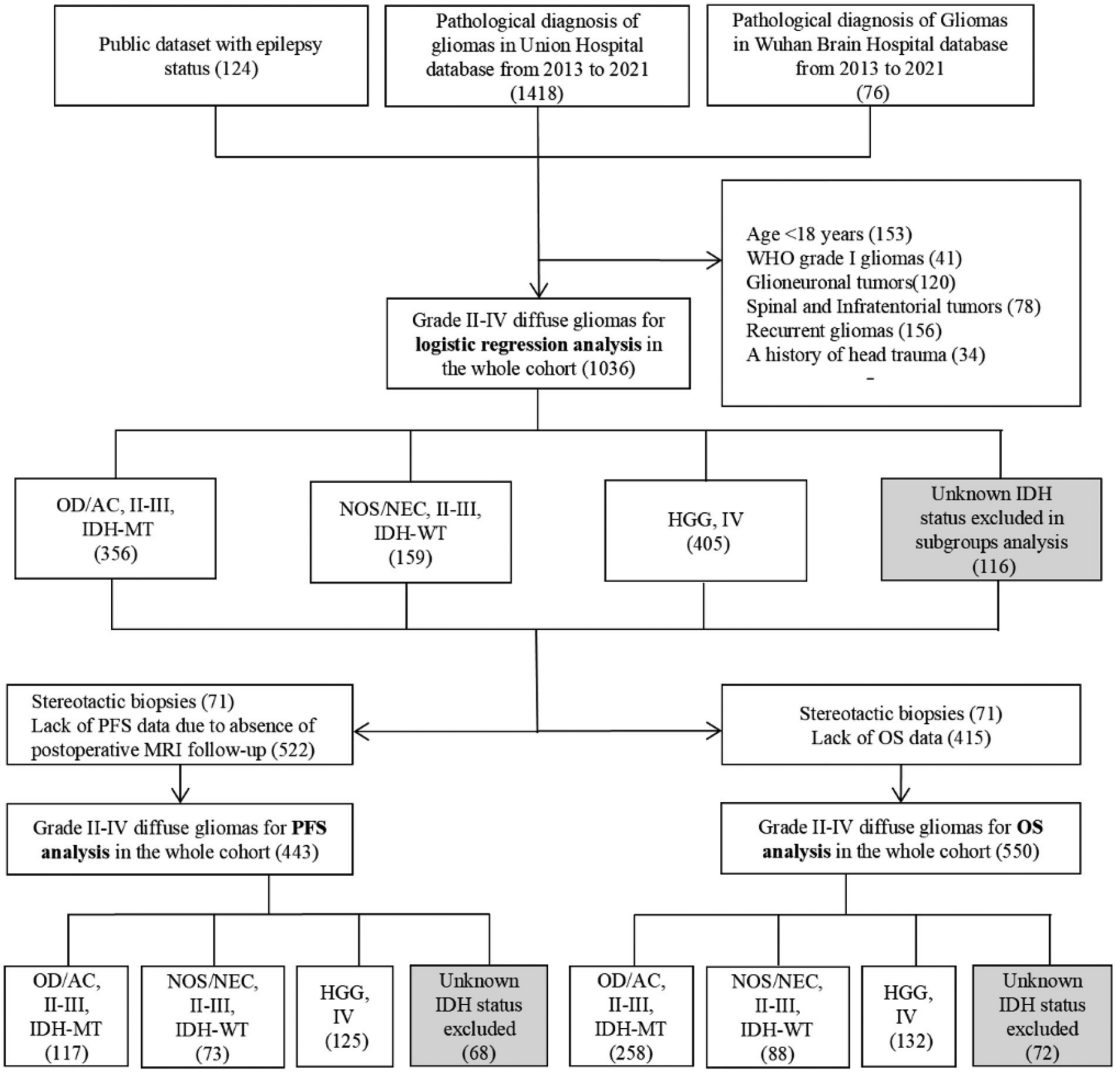
The inclusion and exclusion flowchart. OD/AC: oligodendroglioma/astrocytoma, grade II–III, IDH‐MT; NOS/NEC: not otherwise specified or not elsewhere classified, grade II–III, IDH‐WT; HGG: high‐grade gliomas, grade IV.

This cohort, which includes data from three institutions—Institutes 1 and 2, comprising consecutive sequences, and Institute 3, a public dataset—ultimately consists of 1036 newly diagnosed WHO grade II–IV diffuse gliomas (Table S1). The study's inclusion flowchart is presented in Figure 1. Institution 1 had a lower proportion of IDH‐mutant (IDH‐MT) cases compared to the other two institutions (41.0% vs. 75.0% and 77.4%). In aggregate, gliomas were classified as grades II (33.5%, 343/1025), III (26.9%, 277/1025), and IV (39.6%, 405/1025). The incidence of tumor‐related epilepsy (TRE) varied across the grades, occurring in 46.1% (158/343) of grade II, 32.1% (89/277) of grade III, and 16.5% (67/405) of grade IV gliomas. The occurrence of IDH‐MT was 53.9% (205/273) in grade II, 39.7% (11/243) in grade III, and 6.3% (24/286) in grade IV gliomas. According to histological grade and IDH mutation status, the cohort was categorized into three groups: lower‐grade oligodendroglioma/astrocytoma (OD/AC, grades II–III, IDH‐MT), glioma not otherwise specified or not elsewhere classified (NOS/NEC, grades II–III, IDH‐WT), and high‐grade gliomas (HGG, grade IV). The TRE incidence was 44.4% (158/356) in the OD/AC group (*N* = 356; M/F: 197/159; median age [IQR], 44 years [36–52]), 25.8% (41/159) in the NOS/NEC group (*N* = 159; M/F: 92/67; median age [IQR], 52 years [38–60]), and 16.5% (67/405) in the HGG group (*N* = 405; M/F: 244/161; median age [IQR], 55 years [46–62]).

### 3.2 Postoperative Management

Treatment decisions were made through the collaborative efforts of a multidisciplinary tumor board consisting of neurosurgeons, neuro‐oncologists, and radiation oncologists. Among patients with postoperative data available, 54 patients (13.2%) did not receive adjuvant treatment after surgery, 53 patients (13.0%) received radiotherapy alone, 55 patients (13.5%) were treated with chemotherapy alone, and 246 patients (60.3%) received combination radiotherapy and chemotherapy. Chemotherapy regimens consisted of temozolomide or a regimen of procarbazine, lomustine, and vincristine (PCV). Thirty‐five patients had incomplete postoperative management records. Radiotherapy was delivered via conformal‐field techniques with a median dose of 56 Gy (50–60 Gy) in conventional fractions. All patients with preoperative seizures were treated with anti‐seizure medications.

### 3.3 Risk Factors for TRE

Clinico‐radiologic and molecular characteristics are shown with univariate analyses in Table 1 and multivariate analyses in Table 2. In the OD/AC group, univariate analysis showed that younger age, oligodendroglioma subtype, and tumors not crossing the midline were significantly associated with TRE. However, multivariate analysis identified age as the only independent factor linked to TRE (OR = 0.961; 95% CI, 0.934–0.987; *p* = 0.004). Within the NOS/NEC group, univariate analysis demonstrated significant associations between younger age, female sex, tumor extension, and ATRX‐negative expression and TRE. Multivariate analysis indicated that the absence of deep structure involvement on imaging was the sole independent factor associated with TRE (OR = 0.115; 95% CI, 0.04–0.34; *p* < 0.001). In the HGG group, univariate analysis found that TRE was significantly related to frontal or parietal lobe location, tumor extension, and ATRX‐negative expression. Similar to the NOS/NEC group, multivariate analysis showed that the lack of deep structure involvement remained the only independent variable associated with TRE (OR = 0.176; 95% CI, 0.07–0.48; *p* < 0.001). Across all groups, there were no significant associations between TRE occurrence and 1p/19q codeletion, MGMT promoter methylation, TERT promoter mutation, or EGFR amplification (Tables 1 and 2).

**TABLE 1:** Clinico‐radiological and molecular characteristics of LGG, NEC, and HGG groups.

Variables	OD/AC, IDH‐MT (*N* = 356)			NOS/NEC, IDH‐WT (*N* = 159)			HGG (*N* = 405)		
	Epilepsy (%)	No epilepsy (%)	*p* value	Epilepsy (%)	No epilepsy (%)	*p* value	Epilepsy (%)	No epilepsy (%)	*p* value
No. of patients	158 (44.4)	198 (55.6)		41 (25.8)	118 (74.2)		67 (16.5)	338 (83.5)	
Median age, year (IQR)	40 (34–49)	45.5 (38.25–53.75)	**<0.001**	43 (33–53)	54 (34–54)	**0.007**	51 (44–61)	56 (46–62)	0.101
Gender			0.24			**0.002**			0.135
Male	93 (58.9)	104 (52.5)		15 (36.6)	77 (65.3)		46 (68.7)	198 (58.6)	
Female	65 (41.1)	94 (47.5)		26 (63.4)	41 (34.7)		21 (31.3)	140 (41.4)	
Pathologic diagnosis			**0.037**			0.247			0.204
Oligodendroglioma with oligoastroglioma	103 (66.9)	110 (55.8)		16 (40.0)	35 (29.9)		—	—	
Astrocytomas	51 (33.1)	87 (44.2)		24 (60.0)	82 (70.1)		2 (2.9)	28 (7.2)	
Glioblastoma	—	—		*N*	*N*		65 (97.1)	319 (92.8)	
Grade (WHO 2016)			0.126			0.584			—
2	97 (61.4)	108 (54.5)		19 (46.3)	48 (40.7)		—	—	
3	61 (38.6)	90 (45.5)		22 (53.7)	70 (59.3)		—	—	
4	—	—					—	—	
Midline crossing			**0.018**			0.285			0.092
No	83 (83.8)	116 (70.3)		21 (72.4)	69 (60.5)		39 (73.6)	197 (60.8)	
Yes	16 (16.2)	49 (29.7)		8 (27.6)	45 (39.5)		14 (26.4)	127 (39.2)	
Main tumor location			0.298			0.450			**0.043**
Temporal	20 (20.2)	37 (22.4)		10 (34.5)	40 (35.1)		15 (25.4)	90 (27.2)	
Frontal	60 (65.7)	105 (63.6)		12 (41.4)	41 (36.0)		20 (33.9)	131 (39.6)	
Parietal	4 (4.04)	12 (7.3)		4 (13.8)	9 (7.9)		19 (32.2)	56 (16.9)	
Insula	9 (9.09)	8 (4.9)		1 (3.4)	4 (3.5)		3 (5.1)	10 (3.0)	
Occipital	0 (0)	3 (1.8		0 (0)	12 (10.5)		2 (3.4)	39 (11.8)	
Deep structures (Corpus callosum et al)	1 (1.01)	0 (0)		2 (6.9)	8 (7.0)		0 (0)	5 (1.5)	
Tumor extension			0.125			**<0.001**			**<0.001**
1 lobe	44 (44.4)	66 (40)		13 (44.8)	17 (14.9)		13 (6.1)	20 (6.2)	
≥ 2 lobes	24 (24.2)	28 (17.0)		6 (20.7)	18 (15.8)		16 (30.2)	68 (20.9)	
1 or > 1 lobes with involvement of deep structures	31 (31.3)	71 (43)		10 (34.5)	79 (69.3)		24 (45.3)	238 (72.9)	
Molecular data									
Ki‐67			0.267			0.137			0.269
Low expressed	69 (72.6)	104 (65.4)		17 (60.7)	48 (43.2)		4 (9.1)	13 (4.7)	
High expressed	26 (27.4)	55 (34.6)		11 (39.3)	63 (56.8)		40 (90.9)	262 (95.3)	
P53			0.219			0.421			0.871
Low expressed	97 (63.8)	105 (56.8)		26 (65.0)	82 (72.6)		18 (39.1)	101 (40.9)	
High expressed	55 (36.2)	80 (43.2)		14 (35.0)	31 (27.4)		28 (60.9)	146 (59.1)	
ATRX			0.899			**0.017**			**0.049**
Positive	90 (71.4)	127 (70.2)		26 (81.3)	104 (95.4)		34 (81.0)	215 (91.5)	
Negative	36 (28.6)	54 (29.8)		6 (18.7)	5 (4.6)		8 (19.0)	20 (8.5)	
IDH1			—			—			0.549
Wild type	—	—		—	—		41 (89.1)	221 (92.1)	
Mutation	—	—		—	—		5 (10.9)	19 (7.9)	
1p/19q codeletion			1			—			—
1p/19q—intact	24 (28.9)	23 (29.5)		—	—		—	—	
1p/19q—codeleted	59 (71.1)	55 (70.5)		—	—		—	—	
pMGMT methylation			1			1			1
pMGMT—unmethylated	9 (45.0)	33 (47.8)		2 (66.7)	8 (66.7)		3 (75.0)	21 (65.6)	
pMGMT—methylated	11 (55.0)	36 (52.2)		1 (33.3)	4 (33.3)		1 (25.0)	11 (34.3)	
pTERT mutation			0.596			1			0.131
pTERT—intact	11 (30.5)	8 (23.5)		4 (66.7)	6 (60.0)		0 (0)	8 (47.1)	
pTERT—mutant	25 (69.4)	26 (76.5)		2 (33.3)	4 (40.0)		4 (1.0)	9 (52.9)	
EGFR amplification			0.242			1			1
EGFR—intact	3 (1.0)	9 (52.9)		0 (0)	2 (66.7)		1 (1.0)	4 (44.4)	
EGFR—amplified	0 (0)	8 (47.1)		0 (0)	1 (33.3)		0 (0)	5 (55.6)	

IQR: interquartile range; Corpus callosum et al: either corpus callosum, basal ganglia or brainstem; OD/AC: oligodendroglioma/astrocytoma, grade II–III, IDH‐MT; NOS/NEC: not otherwise specified or not elsewhere classified, grade II–III, IDH‐WT; HGG: high‐grade gliomas, grade IV.

The bold values mean the statistical significance less than 0.05.

**TABLE 2:** Multivariate predictors of preoperative tumor‐related epilepsy.

Variables	OD/AC, IDH‐MT (*N* = 259)		NOS/NEC, IDH‐WT (*N* = 135)		HGG (*N* = 267)	
	OR (CI 95%)	*p* value	OR (CI 95%)	*p* value	OR (CI 95%)	*p* value
Age	0.961 (0.934–0.987)	**0.004**	0.972 (0.939–1.005)	0.105	0.997 (0.966–1.03)	0.809
Female	—	—	2.402 (0.921–6.497)	0.076	—	*N*
Astrocytomas	0.914 (0.539–1.547)	0.738	—	—	—	*N*
Midline crossing	0.539 (0.276–1.015)	0.062	—	—	—	*N*
Tumor extension ≥ 2 lobes	—	—	0.344 (0.09–1.205)	0.104	0.343 (0.109–1.068)	0.064
Tumor extension with involvement of deep structures	—	—	0.115 (0.036–0.343)	**<0.001**	0.176 (0.066–0.479)	**<0.001**
ATRX negative	—	—	3.960 (0.780–18.967)	0.084	2.800 (0.855–8.448)	0.074

Deep structures: either corpus callosum, basal ganglia or brainstem. OD/AC: oligodendroglioma/astrocytoma, grade II–III, IDH‐MT; NOS/NEC: not otherwise specified or not elsewhere classified, grade II–III, IDH‐WT; HGG: high‐grade gliomas, grade IV.

### 3.4 Recurrence Characteristics

In the progression‐free survival (PFS) analysis, 469 patients were excluded from the original cohort due to the absence of postoperative MRI follow‐up at their original institutions. Consequently, 443 patients (M/F: 253/190; median age [IQR]: 47 years [38–56]) from Institutions 1 and 2 were included in the PFS analysis. The average and maximum follow‐up durations from the time of initial surgery were 15.7 months and 113.5 months, respectively, with a median PFS of 25.0 months (95% CI: 19.8–33.3) for the entire cohort. The 1‐year and 3‐year disease‐free survival rates were 67.0% and 38.4%, respectively. At the time of analysis, 193 patients (43.6%) had experienced recurrence, while 250 patients (56.4%) had either not shown disease progression or lost to follow‐up at the date of analysis (Table S2). The median PFS across glioma subgroups was 45 months (95% CI: 35.6–65.0) for OD/AC, 16.5 months (95% CI: 11.3–37.1) for NOS/NEC, and 8.5 months (95% CI: 7.4–9.9) for HGG.

Of the 193 patients with recurrence, 55 (28.5%) underwent repeat surgical resection. At the time of recurrence, 3.6%, 12.7%, and 27.3% of patients with grades II, III, and IV gliomas, respectively, retained their original histological grade; 30.9% of grade II–III were upgraded to grade IV, and 23.6% of grade II were upgraded to grade III. The median time to recurrence was not significantly different between patients with and without histological deterioration at the second surgery (36.0 months vs. 29.5 months, *p* = 0.180). However, patients initially diagnosed with grade IV gliomas had a shorter median time to recurrence (10.2 months; 95% CI: 7.1–22.4). Tumor‐related epilepsy (TRE) was the only factor significantly associated with maintaining histological grade at recurrence (HR = 0.094; 95% CI: 0.01–0.64, *p* = 0.005), indicating that patients without preoperative TRE were more likely to exhibit aggressive tumor progression at recurrence (Table S3).

### 3.5 Predictors of PFS in OD/AC, NOS/NEC, and HGG Groups

**FIGURE 2:**
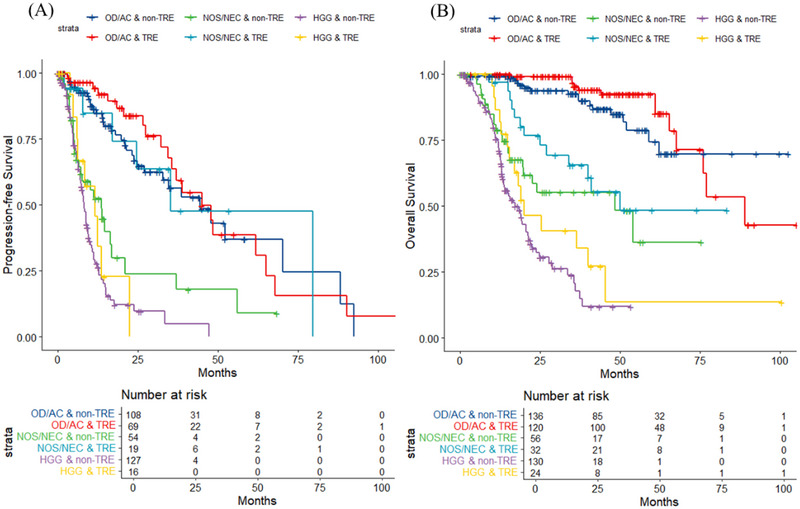
Progression‐free survival (A) and overall survival (B) according to gliomas patients with tumor‐related epilepsy (TRE) and non‐TRE in OD/AC, NOS/NEC and HGG groups. OD/AC: oligodendroglioma/astrocytoma, grade II–III, IDH‐MT; NOS/NEC: not otherwise specified or not elsewhere classified, grade II–III, IDH‐WT; HGG: high‐grade gliomas, grade IV.

Patients without TRE at diagnosis had a significantly shorter median PFS of 14.8 months (95% CI: 12.8–21.1) compared to those with TRE, whose median PFS was 42.5 months (95% CI: 34.5–65.0) (HR = 0.436; 95% CI: 0.31–0.61, *p* < 0.001) (Figure 2). After adjusting for clinical factors such as grade, age, TRE status, radiographic features, tumor volume, and treatment modalities, multivariate analysis identified IDH mutation (HR = 0.438; 95% CI: 0.25–0.77, *p* = 0.004), gross total resection (HR = 0.174; 95% CI: 0.07–0.41, *p* < 0.001), and adjuvant therapy (HR = 0.325; 95% CI: 0.15–0.69, *p* = 0.003) as independent prognostic factors for improved survival (Table S3).

**FIGURE 3:**
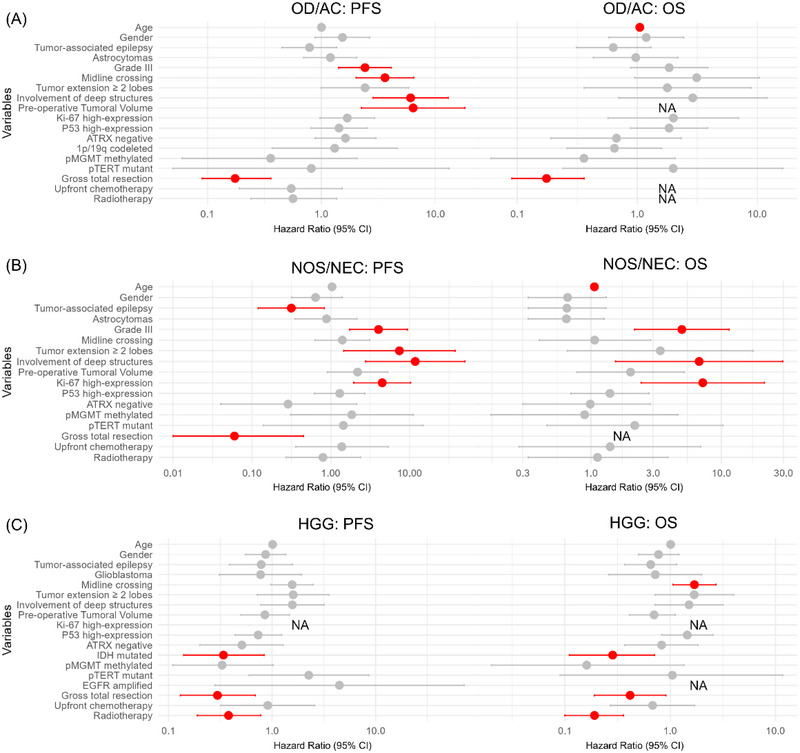
Forest plot illustrating the PFS and OS of univariate survival analyses for the OD/AC (A), NOS/NEC (B), and HGG (C) groups. Deep structures: either corpus callosum, basal ganglia or brainstem; OD/AC: oligodendroglioma/astrocytoma, grade II–III, IDH‐MT; NOS/NEC: not otherwise specified or not elsewhere classified, grade II–III, IDH‐WT; HGG: high‐grade gliomas, grade IV; PFS: progression‐free survival; OS: overall survival; NA: data on subgroup occurrence outcomes were insufficient to calculate median survival.

For the OD/AC group (*N* = 177), those with TRE at diagnosis had a similar median PFS of 45.0 months (95% CI: 35.6–90.0) compared to 44.4 months (95% CI: 32.8–NA) in those without TRE. Multivariate analysis revealed that grade III gliomas, tumors extending across multiple lobes, and deep brain structure involvement were significantly associated with poorer PFS (Table 3) (Figure 3A). In the NEC group (*N* = 73), patients with TRE exhibited a longer median PFS of 35.2 months (95% CI: 24.6–NA) compared to 13.6 months (95% CI: 7.2–37.1) in those without TRE (*p* = 0.02), though TRE was not an independent prognostic factor. Multivariate analysis highlighted that tumor extension across multiple lobes and deep brain structure involvement were significantly associated with shorter PFS (Table 3) (Figure 3B). Gross total resection was excluded from multivariate analysis due to a large coefficient; however, patients who underwent GTR had significantly longer median PFS (67.8 months [95% CI: 56.0–NA] vs. 13.6 months [95% CI: 7.6–21]) (*p* = 0.006), underscoring the role of GTR as a favorable prognostic indicator in NEC gliomas (Tables 3 and S5). In the HGG group (*N* = 143), patients with TRE had a longer median PFS of 11.7 months (95% CI: 6.3–NA) compared to 8.2 months (95% CI: 7.1–9.6) in those without TRE, though the difference was not statistically significant (*p* = 0.494). Both univariate and multivariate analyses confirmed that IDH mutation (HR = 0.324; 95% CI: 0.12–0.86, *p* = 0.023), gross total resection (HR = 0.297; 95% CI: 0.12–0.72, *p* = 0.007), and adjuvant therapy (HR = 0.671; 95% CI: 0.49–0.91, *p* = 0.011) were independently associated with favorable outcomes (Table 3) (Figure 3C).

**TABLE 3:** Multivariate analysis of prognostic factors for PFS and OS of OD/AC, NOS/NEC, and HGG groups.

		PFS			OS	
	*N*	HR (CI 95%)	*p* value	*N*	HR (CI 95%)	*p* value
**OD/AC group**					—	—
No. of patients	177			258	—	—
Median age, y (IQR)	43 (34–51)			43 (35–52)	—	—
Grade (2016 WHO CNS)					—	—
2	119	1		133	—	—
3	58	2.634 (1.26–5.51)	**0.010**	125	—	—
Tumor extension					—	—
1 lobe	74	1		70	—	—
≥ 2 lobes	35	4.604 (1.13–18.83)	**0.034**	30	—	—
1 or > 1 lobes with involvement of deep structures	68	1.844 (0.39–8.75)	0.441	66	—	—
Extent of resection (EOR)					—	—
Nongross total resection (non‐GTR)	101			99	—	—
Gross total resection (GTR)	76	0.273 (0.09–0.87)	**0.029**	67	—	—
**NOS/NEC group**						
No. of patients	73			88		
Median age, year (IQR)	52 (42–61)	1.036 (0.99–1.08)	0.102	52 (39.75–61)	1.079 (1.02–1.14)	**0.006**
Tumor extension						
1 lobe	20	1		19	1	
≥ 2 lobes	14	11.483 (1.26–104.53)	**0.03**	14	1	
1 or > 1 lobes with involvement of deep structures	39	20.412 (2.15–193.41)	**0.009**	38	1.736 (1.08–2.77)	**0.020**
Ki‐67						
Low expressed	34	1		34		
High expressed	37	0.591 (0.16–2.12)	0.418	36	0.851 (0.18–3.88)	0.834
Extent of resection (EOR)						
Nongross total resection (non‐GTR)	59			60		
Gross total resection (GTR)	16			13		
**HGG group**						
No. of patients	143			154		
IDH						
Wild type	125	1		132	1	
Mutation	10	0.324 (0.12–0.86)	**0.023**	14	0.432 (0.15–1.23)	0.115
Extent of resection (EOR)						
Nongross total resection (non‐GTR)	127	1		124	1	
Gross total resection (GTR)	16	0.297 (0.12–0.72)	**0.007**	15	0.545 (0.23–1.29)	0.169
Postoperative management						
Observation with MRI	23	1		22	1	
Upfront chemotherapy alone	11	1		11	1	
Radiotherapy or radio‐chemotherapy	93	0.671 (0.49–0.91)	**0.011**	90	0.475 (0.35–0.64)	**<0.001**

Deep structures: either corpus callosum, basal ganglia or brainstem; OD/AC: oligodendroglioma/astrocytoma, grade II–III, IDH‐MT; NOS/NEC: not otherwise specified or not elsewhere classified, grade II–III, IDH‐WT; HGG: high‐grade gliomas, grade IV; PFS: progression‐free survival; OS: overall survival.

### 3.6 Overall Survival Characteristics

A total of 550 patients with available overall survival (OS) data were included from Institution 1 and the public dataset. Of these, 164 patients had died by the time of follow‐up, while 385 patients were either alive or lost to follow‐up. The OS cohort comprised 345 males and 205 females, with a median age of 46 years (IQR: 37–56), and included a higher proportion of IDH‐mutant gliomas compared to the PFS cohort (49.5% vs. 42.2%). The median OS for the entire cohort was 77.0 months (95% CI: 67.5–NA), with 3‐year and 5‐year OS rates of 71.7% and 60.5%, respectively. The median OS for NOS/NEC and HGG subgroups were 50 months (95% CI: 27.0–NA) and 18.5 months (95% CI: 15.0–21.4), respectively (Table S2).

### 3.7 Predictors of OS in OD/AC, NOS/NEC, and HGG Groups

TRE at diagnosis was not a significant independent predictor of longer OS across the entire cohort (HR = 0.349; 95% CI: 0.06–1.89, *p* = 0.223), after adjusting for variables such as age, tumor grade, midline crossing, tumor extension, volume, IDH mutation, Ki67 and P53 expression, extent of resection, and combined chemoradiotherapy (Table S3).

In the OD/AC group (*N* = 258), univariate analysis showed that older age was the only significant negative prognostic factor (HR = 1.049; 95% CI: 1.01–1.09, *p* = 0.007) (Table 3) (Figure 2A). In the NOS/NEC group (*N* = 88), the median OS for patients with TRE was 50.0 months, compared to 48.4 months for those without. Multivariate analysis identified older age and deep brain structure involvement as significant independent predictors of worse OS (Table 3) (Figure 2B). In the HGG group (*N* = 154), the median OS for patients with TRE was 20.0 months, compared to 17.2 months for those without. Radiotherapy with or without chemotherapy was the only factor significantly associated with longer OS (HR = 0.475; 95% CI: 0.35–0.64) (Table 3) (Figure 3C).

## 4 Discussion

Since the release of the fifth edition of the WHO classification for central nervous system (CNS) tumors, significant changes in the categorization of gliomas have provided deeper insights into their characteristics and prognosis. Concurrently, translational research has demonstrated a bidirectional relationship between glioma growth and hyperexcitability, although the relationship between these glioma‐neuron interactions and tumor‐related epilepsy (TRE) remains poorly understand (Winkler 2022). In this study of newly diagnosed glioma, including two Chinese cohorts and a publicly available Western cohort with TRE data, stratified into prespecified prognostic subgroups, TRE was not a strong independent predictor of survival after controlling for clinical and molecular tumor features. Notably, even in the absence of tumor sequencing in many patients, adequate prognostic separation was achieved to clarify the negligible impact of TRE on survival outcomes. These findings suggest that the prognostic relevance of preoperative TRE is likely driven by underlying tumor biology and associated clinical factors, rather than the presence of seizures specifically.

### 4.1 Risk Factors

The incidence of TRE varies significantly between patients with IDH‐mutant (IDH‐MT) and IDH‐wild‐type (IDH‐WT) gliomas, ranging from 41.5% to 77.1% for IDH‐MT gliomas and 16.8% to 44.6% for IDH‐WT gliomas in previous studies (Pallud et al. 2024; Drumm et al. 2023; Guo et al. 2023; Ricklefs et al. 2022; Carstam et al. 2022; Osada et al. 2021; Yuan et al. 2020; Yu et al. 2019; Duan et al. 2018; Neal et al. 2018; Chen et al. 2017; Zhou et al. 2016; Berendsen et al. 2016; Toledo et al. 2015; Wychowski et al. 2013; Bauchet et al. 2010; Lote et al. 1998; Bruno et al. 2024; Pallud et al. 2014). Our study reveals a similar trend, with decreasing TRE incidence across the three glioma subgroups. These findings align with prior research in Chinese glioma populations (Guo et al. 2023; Duan et al. 2018), though TRE rates tend to be higher in Western populations (Pallud et al. 2024; Drumm et al. 2023; Neal et al. 2018). In our study, we identified age as an independent risk factor for TRE in patients with LGG, IDH‐MT gliomas. This may be partially explained by the fact that younger patients have more unstable neuronal networks and neurotransmitter levels, leading to increased neuronal excitability and susceptibility to abnormal discharges (Chen et al. 2018). Additionally, preclinical studies suggest that D‐2‐hydroxyglutarate (D‐2HG), a metabolite produced by IDH‐mutant gliomas, can excite neurons by mimicking glutamate or activating the mTOR signaling pathway (Drumm et al. 2023; Chen et al. 2017; Mazzucchi et al. 2022). This mechanism may contribute to the higher incidence of TRE with elevated tissue 2HG levels in IDH‐MT gliomas compared to IDH‐WT (Duan et al. 2018). It is worth investigating whether younger patients with IDH‐MT gliomas have a greater capacity to produce 2HG. In contrast, tumor extension emerged as an independent predictor of TRE in NOS/NEC and HGG patients. IDH‐WT gliomas, including glioblastoma, have a distinct driver mutations and genetic profiles compared to IDH‐MT gliomas, likely contributing to multiple interacting peritumoral molecular alterations that determine epileptogenicity (Soeung et al. 2024; Tobochnik et al. 2024; Yu et al. 2020). Loss of α‐thalassemia/mental retardation syndrome X‐linked (ATRX‐negative) may be a secondary factor. As a chromatin remodeling protein, ATRX mutations can lead to dysregulated gene expression, affecting neuronal excitability and potentially increasing the risk of epilepsy (Aiello et al. 2022). Our study showed that ATRX negative was significantly associated with TRE in NOS/NEC and HGG patients in univariate logistic regression analysis. However, this association was not significant in multivariate logistic regression analysis, likely due to the small number of ATRX‐negative cases in the IDH‐WT group. Finally, tumor extension into deep structures may serve as a stronger predictor of the absence of preoperative epilepsy occurrence in patients with IDH‐WT gliomas.

### 4.2 Seizures and Prognosis

The first part of our study revealed a higher prevalence of seizures in glioma patients with relatively favorable prognoses. Similarly, the prognostic role of tumor‐related epilepsy (TRE) in glioma patients has garnered significant attention. Previous studies have primarily investigated the relationship between preoperative TRE and overall survival (OS) in adult diffuse gliomas, though findings remain inconclusive. For instance, one study identified preoperative seizures as an independent predictor of significantly prolonged OS in patients with grade III–IV gliomas, but not in those with grade II gliomas (Lote et al. 1998; Fairclough et al. 2024). In contrast, another study reported that TRE was an independent predictor of prolonged OS in grade II gliomas. (Pallud et al. 2014) and Zhou et al. (2016) found preoperative TRE to be associated with prolonged OS in grade II–III gliomas (109 months vs. 79 months) in a Chinese cohort. Chen et al.’s (2017) analysis across three institutions found no difference in median survival between IDH1‐mutant gliomas with or without seizures, but demonstrated extended median OS in IDH‐WT gliomas in three institution cohorts (from 7.6 months to 12.6 months and 13.6 months to 18.6 months, respectively). Multiple studies have also reported a statistically significant association between TRE and prolonged median OS in glioblastoma (GBM) patients (Berendsen et al. 2016; Toledo et al. 2015; Pallud et al. 2014; Salvati et al. 2020; Bianconi et al. 2024), though some findings indicate this correlation as marginally nonsignificant (Flanigan et al. 2017; Wychowski et al. 2013). Taken together, the findings from this study support different risks and mechanisms of epileptogenicity between diffuse glioma pathologies but that the favorable prognostic association of TRE appears to be largely driven by tumor biology. Nonetheless, mechanisms accounting for a potential independent effect deserve further investigation.

The impact of preoperative tumor‐related epilepsy (TRE) on progression‐free survival (PFS) in adult diffuse gliomas has not been thoroughly investigated. Pallud et al. (2014) identified preoperative TRE as an independent predictor of significantly prolonged PFS in grade II gliomas, though their analysis did not account for IDH mutation status. Santos‐Pinheiro et al. reported similar findings, observing significant differences only in univariate analysis (Bianconi et al. 2024; Santos‐Pinheiro et al. 2019). Another study on grade II–III gliomas showed a favorable trend, but without significant correlation (Zhou et al. 2016). More recent research on IDH‐MT low‐grade gliomas (LGG) did not identify TRE as a significant predictor of PFS; however, the absence of seizures following adjuvant therapy correlated with longer median PFS (Bruno et al. 2024). A study of 587 adult patients with grade III–IV gliomas found preoperative TRE to be a significant predictor of PFS in univariate survival analysis alone (Yu et al. 2019). These studies are in line with our findings, which further specify this trend in grade II–III, IDH‐wild‐type (IDH‐WT) gliomas and seen only in univariate analysis. This may be due to their tendency to invade deeper brain structures, which accelerates malignant progression.

### 4.3 Prognostic Factors in LGG, NOS/NEC, and HGG Groups

In this study, the median PFS for the LGG, IDH‐MT group was 45 months, slightly shorter than the results by Bruno et al. (2024). Nevertheless, we similarly observed that complete surgical resection significantly prolongs median PFS. Another study of LGG patients with epilepsy reported a median overall survival (OS) of 79.3 months, aligning with our cohort (median OS: 89 months) (Mazzucchi et al. 2022). Prognosis in IDH‐WT astrocytomas varies significantly depending on other genetic mutations, such as epidermal growth factor receptor (EGFR) amplification, whole chromosome 7 gain and whole chromosome 10 loss (+7/−10), and telomerase reverse transcriptase (TERT) promoter mutations. One study reported that the median OS for IDH‐WT grade II diffuse gliomas that meet the molecular definition of glioblastoma (GBM) was 42 months, compared to 57‐month median OS for IDH‐WT grade II diffuse gliomas that do not meet the molecular GBM definition. IDH‐WT grade III diffuse gliomas also had a shorter median OS (17 months vs. 19 months), which corroborates our findings (Berzero et al. 2021). Ruda et al. analyzed IDH‐WT low‐grade gliomas and IDH‐WT GBM and found that both the median PFS and OS for IDH‐WT low‐grade gliomas were significantly longer than those for IDH‐WT GBM. EGFR amplification and TERT promoter mutations also shortened the PFS and OS of IDH‐WT grade II diffuse gliomas (Bianconi et al. 2024; Rudà et al. 2022). However, Berzero et al. (2021) argued that TERT promoter mutations alone in IDH‐WT grade II diffuse gliomas do not impact survival. Thus, the prognosis for IDH‐WT low‐grade diffuse gliomas remains unclear. Our findings identified independent radiographic characteristics that carry prognostic value: invasion of deep brain structures, rather than tumor size, significantly affects the prognosis of IDH‐WT low‐grade diffuse gliomas. In the high‐grade glioma (HGG) group, the incidence of IDH mutations was consistent with previous studies, and their positive prognostic impact was similarly observed (Chen et al. 2017; Wong et al. 2021). Adjuvant therapy significantly influenced the prognosis in the HGG group but had no noticeable effect on the LGG and nonenhancing contrast (NOS/NEC) groups, in line with previous research (Mazzucchi et al. 2022; Wang et al. 2021).

This study has limitations. First, data were collected retrospectively and focused only on preoperative epilepsy, limiting the characterization of seizures and seizure frequency. Previous studies have shown that different types of seizures may have distinct prognostic implications (Kerkhof and Vecht 2013; Fairclough et al. 2024; De La Cerda‐Vargas et al. 2024). Standardized seizure tracking performed prospectively over the course of disease is necessary to improve epilepsy phenotyping. Second, the findings may not be entirely generalizable to all glioma populations, as tumor‐related epilepsy (TRE) incidence is generally higher in Western cohorts compared to the Chinese population studied (Pallud et al. 2024; Drumm et al. 2023; Neal et al. 2018). Third, our center began testing for IDH mutations only after 2016, primarily using immunohistochemistry, resulting in insufficient data for studying the full extent of IDH1 and IDH2 mutations. As a result, some patients lacked comprehensive molecular classification, which may have affected the robustness of our conclusions regarding the relationship between genetic factors and TRE. Furthermore, due to limited resources for next generation sequencing, only a subset of patients had sequencing for IDH1/2 mutations, copy number changes, and molecular GBM variants. A study investigating IDH1 and IDH2 gene mutations in the Chinese population using PCR‐Sanger sequencing revealed a relatively low IDH1 mutation rate, with only 31.6% (74/234) presenting the IDH1 R132H mutation and no other IDH1and IDH2 mutations which aligned with the results reported in another Chinese study (潘 et al. 2013; 黎 et al. 2016). While our study included relatively large glioma NOS/NEC subgroup, nevertheless, the associations with TRE were consistent across subgroups, suggesting that even if some of these tumors were reclassified as either IDH‐MT OD/AC or GBM, it would be unlikely to change the interpretation of these results with respect to the primary hypothesis. Fourth, the extent of resection in our study was determined based on postoperative MRI (2–3 months post‐surgery) rather than volumetric analyses, as recommended by the latest RANO criteria. While this method remains a widely accepted clinical approach, volumetric assessments would provide a more precise quantification of residual tumor burden and its relationship with outcomes. Finally, the follow‐up period was too short to capture sufficient outcomes in IDH‐MT OD/AC patients. Studies with longer follow‐up periods and larger patient cohorts are needed to address this issue.

In conclusion, our findings underscore the importance of stratifying adult diffuse gliomas by pathologic and molecular prognostic subgroups when investigating mechanisms of tumor‐related epilepsy and clinical implications. Seizures result from a complex interaction between tumor cells and surrounding brain tissue, driven by the underlying tumor biology. Future strategies targeting these interactions may improve both seizure control and survival outcomes.

## Author Contributions

**Yao Xiao**: Writing—original draft; writing—review and editing; methodology; conceptualization. **Zhuang Nie**: Methodology; data curation; investigation; writing—review and editing; conceptualization; formal analysis. **Jinsha Huang**: Data curation; methodology; validation; writing—review and editing; conceptualization. **Jie Zhao**: Writing—review and editing; methodology. **Chengjun Dong**: Conceptualization; data curation; writing—original draft. **Yan Zou**: Methodology. **Zikai Li**: Data curation. **Bingqing Yan**: Investigation. **Yue Hu**: Data curation; investigation. **Fan Yang**: Supervision. **Jong Woo Lee**: Writing—review and editing; methodology; supervision. **Alexander P. Lin**: Methodology; writing—review and editing; supervision; validation. **Steven Tobochnik**: Methodology; supervision; writing—review and editing. **Min Zhou**: Methodology; writing—review and editing; supervision; funding acquisition. **Ziqiao Lei**: Methodology; writing—review and editing; supervision; resources; project administration; funding acquisition.

## Conflicts of Interest

The authors have no conflict of interest. None of the authors is current Editor or Editorial Board Member.

## Ethics Statements

The protocol was approved by the Institutional Review Board of Union Hospital and General Hospital of Yangtze River Shipping.

### Peer Review

The peer review history for this article is available at https://publons.com/publon/10.1002/brb3.70510.

## Supporting information



Supporting Information

## Data Availability

The local datasets generated or analyzed during the study are not publicly available because of institutional regulations, but they are available from the corresponding author on reasonable request. The public datasets analyzed in this study were obtained from the POLA Network, retrieved from ArrayExpress at E‐MTAB‐3892.
