# Supplementary material for: Risk Factors and Prognostic Implications of Tumor‐Related Epilepsy in Diffuse Glioma Patients: A Real‐World Multicenter Study
Source: Brain Behav. 2025 May 5;15(5):e70510. doi: 10.1002/brb3.70510 (PMC12050638; doi:10.1002/brb3.70510)
Supplement: Supplementary file 1 — Supporting Information [file BRB3-15-e70510-s001.docx]

Supplementary Table S1. Patient cohort characteristics

| Characteristics | No. of patients | Institute 1 | Institute 2 | Public dataset | *x*^2^ | P value |
| --- | --- | --- | --- | --- | --- | --- |
| Total | 1036 | 877 | 35 | 124 |  |  |
| Median age, y (IQR) | 49 (40-58) | 49 (41-58) | 49 (43-58) | 44 (35-56) | 54 (20) | **0.007** |
| Gender |  |  |  |  | 1.30 | 0.522 |
| Male | 600 (57.9) | 511 (58.3) | 17 (48.6) | 72 (58.1) |  |  |
| Female | 436 (42.1) | 366 (41.7) | 18 (51.4) | 52 (41.9) |  |  |
| Pathologic diagnosis |  |  |  |  | 179.86 | **<0.001** |
| Oligodendroglioma  With Oligoastroglioma | 341 (33.5) | 229 (26.2) | 5 (29.4) | 107 (86.3) |  |  |
| Astrocytomas | 292 (28.7) | 282 (32.2) | 8 (47.1) | 2 (1.6) |  |  |
| Glioblastoma | 384 (37.8) | 365 (41.6) | 4 (23.5) | 15 (12.1) |  |  |
| Grade (WHO 2016) |  |  |  |  | 101.71 | **<0.001** |
| 2 | 343 (33.5) | 300 (34.2) | 12 (50.0) | 31 (25.0) |  |  |
| 3 | 277 (26.9) | 193 (21.9) | 6 (25.0) | 78 (62.9) |  |  |
| 4 | 405 (39.6) | 384 (43.9) | 6 (25.0) | 15 (12.1) |  |  |
| Midline crossing |  |  |  |  | 0.29 | 0.592 |
| No | 615 (68.4) | 593 (68.6) | 22 (62.9) | - |  |  |
| Yes | 284 (31.6) | 271 (31.4) | 13 (37.1) | - |  |  |
| Main tumor location |  |  |  |  | 14.07 | **0.015** |
| Temporal | 239 (26.2) | 224 (25.5) | 15 (42.9) | - |  |  |
| Frontal | 435 (47.7) | 426 (48.6) | 9 (25.7) | - |  |  |
| Parietal | 119 (13.0) | 115 (13.1) | 4 (11.4) | - |  |  |
| Insula | 38 (4.2) | 36 (4.1) | 2 (5.7) | - |  |  |
| Occipital | 60 (6.6) | 58 (6.6) | 2 (5.7) | - |  |  |
| Deep structures (Corpus callosum et al) | 21 (2.3) | 18 (2.1) | 3 (8.6) | - |  |  |
| Tumor extension |  |  |  |  | 0.317 | 0.853 |
| 1 lobe | 221 (24.6) | 221 (25.5) | 10 (28.6) | - |  |  |
| ≥ 2 lobes | 189 (21.0) | 182 (21.0) | 7 (20.0) | - |  |  |
| 1 or > 1 lobes with involvement of deep structures | 490 (54.4) | 472 (53.5) | 18 (51.4) | - |  |  |
| Tumor-related epilepsy |  |  |  |  | 79.68 | **<0.001** |
| No | 721 (69.6) | 655 (74.7) | 22 (62.9) | 44 (35.5) |  |  |
| Yes | 315 (30.4) | 222 (25.3) | 13 (37.1) | 80 (64.5) |  |  |
| Molecular data |  |  |  |  |  |  |
| Ki-67 |  |  |  |  | 3.87 | **0.049** |
| Low expressed | 276 (36.6) | 264 (36.0) | 12 (60.0) | - |  |  |
| High expressed | 478 (63.4) | 470 (64.0) | 8 (40.0) | - |  |  |
| P53 |  |  |  |  | 8.24 | **0.016** |
| Low expressed | 436 (55.1) | 346 (52.7) | 9 (62.3) | 81 (66.4) |  |  |
| High expressed | 356 (44.9) | 310 (47.3) | 5 (37.7) | 41 (33.6) |  |  |
| ATRX |  |  |  |  | 11.43 | **0.003** |
| Positive | 596 (82.2) | 529 (83.3) | 5 (45.5) | 62 (78.5) |  |  |
| Negative | 129 (17.8) | 106 (16.7) | 6 (54.5) | 17 (21.5) |  |  |
| IDH |  |  |  |  | 59.21 | **<0.001** |
| Wild type | 422 (52.6) | 386 (59.0) | 8 (25.0) | 28 (22.6) |  |  |
| Mutation | 380 (47.4) | 268 (41.0) | 16 (75.0) | 96 (77.4) |  |  |
| 1p/19q codeletion |  |  |  |  |  |  |
| 1p/19q-intact | 82 (41.4) | 33 (44.6) | - | 49 (39.5) |  |  |
| 1p/19q-codeleted | 116 (38.6) | 41 (55.4) | - | 75 (60.6) |  |  |
| pMGMT methylation |  |  |  |  | 0.305 | 0.580 |
| pMGMT-unmethylated | 42 (47.2) | 42 (47.2) | - | - |  |  |
| pMGMT-methylated | 47 (52.8) | 47 (52.8) | - | - |  |  |
| pTERT mutation |  |  |  |  | 6.55 | **0.011** |
| pTERT-intact | 37 (34.6) | 23 (48.9) | - | 14 (23.3) | 6 (60.0) |  |
| pTERT-mutant | 70 (65.4) | 24 (51.1) | - | 46 (76.7) | 4 (40.0) |  |
| EGFR amplification |  |  |  |  |  |  |
| EGFR-intact | 12 (60.0) | 12 (60.0) | - | - | - | - |
| EGFR-amplified | 8 (40.0) | 8 (40.0) | - | - |  |  |

Supplementary Table S2. Characteristics of Patients with Malignance and Maintenance Pathological Outcomes at the Second Surgery

|  |  | Maintenance group (%) | Malignancy group (%) | OR (CI 95%) | P Value |
| --- | --- | --- | --- | --- | --- |
| No. of patients | 39 | 9(23.1) | 30(76.9) |  |  |
| Mean±SD age, y |  | 36.8±8.9 | 43.7±9.3 |  | 0.06 |
| Pathologic diagnosis |  |  |  |  |  |
| Oligodendroglioma  With Oligoastroglioma | 17 | 3 (33.3) | 14 (46.7) | 1 |  |
| Astrocytomas | 22 | 6 (66.7) | 16 (53.3) | 0.58 (0.08-3.35) | 0.704 |
| Grade (WHO 2016) |  |  |  |  |  |
| 2 | 19 | 2 (22.2) | 17 (56.7) | 1 |  |
| 3 | 20 | 7 (77.8) | 13 (43.3) | 0.227 (0.02-1.46) | 0.127 |
| Tumor-related epilepsy |  |  |  |  |  |
| No | 25 | 2 (22.2) | 23 (76.7) | 1 |  |
| Yes | 14 | 7 (77.8) | 7 (23.3) | 0.094 (0.01-0.64) | **0.005** |

Supplementary Table S3. Univariate and Multivariate Analysis of Prognostic Factors for PFS and OS of Grade II-IV Adult-type Diffuse Gliomas

|  | PFS | | | | | OS | | | | |
| --- | --- | --- | --- | --- | --- | --- | --- | --- | --- | --- |
|  |  | Univariate |  | Multivariable |  |  | Univariate |  | Multivariable |  |
|  | N | HR (CI 95%) | P value | HR (CI 95%) | P value | N | HR (CI 95%) | P value | HR (CI 95%) | P value |
| No. of patients | 443 |  |  |  |  | 550 |  |  |  |  |
| Median age±IQR, y | 47 (38-56) | 1.033 (1.02-1.05) | **<0.001** | 1.016 (0.99-1.04) | 0.100 | 46 (37-56) | 1.05 (1.04-1.07) | **<0.001** | 1.031 (1.00-1.06) | **0.032** |
| Tumor-related epilepsy |  |  |  |  |  |  |  |  |  |  |
| No | 312 | 1 |  | 1 |  | 345 | 1 |  | 1 |  |
| Yes | 131 | 0.436 (0.31-0.61) | **<0.001** | 0.914 (0.54-1.56) | 0.742 | 205 | 0.401 (0.29-0.59) | **<0.001** | 0.349 (0.06-1.89) | 0.223 |
| Pathologic diagnosis |  |  |  |  |  |  |  |  |  |  |
| Oligodendroglioma  With Oligoastroglioma | 134 | 1 |  | - |  | 240 | 1 |  | - | - |
| Astrocytomas | 166 | 1.72 (1.17-2.53) | **<0.001** | - |  | 161 | 1.8 (1.15-2.81) | **<0.001** | - | - |
| Glioblastoma | 131 | 6.24 (4.19-9.28) | **<0.001** | - |  | 146 | 9.18 (6.22-13.5) | **<0.001** | - | - |
| Grade (WHO 2016) |  |  |  |  |  |  |  |  |  |  |
| 2 | 189 | 1 |  | 1 |  | 210 | 1 |  | 1 |  |
| 3 | 111 | 2.94 (2.0-4.32) | **<0.001** | 1 |  | 186 | 3.13 (1.92-5.11) | **<0.001** | 3.226(0.69-15.06) | 0.136 |
| 4 | 143 | 8.54 (5.81-12.6) | **<0.001** | 1.32 (0.99-1.76) | 0.060 | 154 | 15.1 (9.41-24.3) | **<0.001** | 4.854 (0.92-26.0) | 0.062 |
| Midline crossing |  |  |  |  |  |  |  |  |  |  |
| No | 324 | 1 |  | 1 |  | 312 | 1 |  | 1 |  |
| Yes | 119 | 1.884 (1.39-2.56) | **<0.001** | 1.423 (0.90-2.29) | 0.145 | 114 | 1.658 (1.14-2.41) | **0.008** | 1.476 (0.69-3.16) | 0.317 |
| Tumor extension |  |  |  |  |  |  |  |  |  |  |
| 1 lobe | 138 | 1 |  | 1 |  | 133 | 1 |  |  |  |
| ≥ 2 lobes | 89 | 2.55 (1.60-4.70) | **<0.001** | 2.349 (0.94-5.85) | 0.067 | 83 | 2.71 (1.45-5.01) | **0.002** | 1.362 (0.39-4.92) | 0.631 |
| 1 or > 1 lobes with involvement of deep structures | 216 | 4.87 (3.23-7.24) | **<0.001** | 1.899 (0.76-4.76) | 0.171 | 210 | 5.10 (2.97-8.77) | **<0.001** | 1.304 (0.37-4.64) | 0.678 |
| Median pre-operative Tumoral Volume computed on T2-weighted images, cm^3^ (IQR) | 96.61 (48.83-159.8) |  |  |  | 96.15 (48.38162.3) |  |  |  |  |  |
| >100 cm^3^ | 161 | 1 |  | 1 |  | 158 | 1 |  | 1 |  |
| ≤100 cm^3^ | 156 | 3.15 (2.15-4.62) | **<0.001** | 1.174 (0.70-1.96) | 0.538 | 146 | 2.54 (1.60-4.02) | **<0.001** | 0.721 (0.34-1.56) | 0.405 |
| Molecular data |  |  |  |  |  |  |  |  |  |  |
| Ki-67 |  |  |  |  |  |  |  |  |  |  |
| Low expressed | 159 | 1 |  | 1 |  | 151 | 1 |  | 1 |  |
| High expressed | 223 | 3.612 (2.48-5.25) | **<0.001** | 1.58 (0.81-3.08) | 0.180 | 220 | 8.162 (4.36-15.28) | **<0.001** | 6.315 (1.58-25.22) | **0.009** |
| P53 |  |  |  |  |  |  |  |  |  |  |
| Low expressed | 182 | 1 |  | - | - | 259 | 1 |  |  |  |
| High expressed | 159 | 1.412 (1.02-1.96) | **0.038** | - | - | 198 | 1.96 (1.38-2.79) | **<0.001** | 0.919 (0.50-1.67) | 0.781 |
| ATRX |  |  |  |  |  |  |  |  |  |  |
| Positive | 255 | 1 |  | - | - | 314 | 1 |  | - | - |
| Negative | 67 | 0.674 (0.43-1.04) | 0.077 | - | - | 84 | 0.553 (0.31-0.99) | **0.047** | - | - |
| IDH |  |  |  |  |  |  |  |  |  |  |
| Wild type | 198 | 1 |  | 1 |  | 220 | 1 |  |  |  |
| Mutation | 187 | 0.23 (0.17-0.32) | **<0.001** | 0.438 (0.25-0.77) | **0.004** | 272 | 0.11 (0.07-0.16) | **<0.001** | 0.130 (0.04-0.39) | **<0.001** |
| 1p/19q codeletion |  |  |  |  |  |  |  |  |  |  |
| 1p/19q-intact | 32 | 1 |  |  |  | 81 | 1 |  |  |  |
| 1p/19q-codeleted | 34 | 1.093 (0.36-3.3) | 0.874 |  |  | 109 | 0.233 (0.12-0.44) | **<0.001** |  |  |
| pMGMT methylation |  |  |  |  |  |  |  |  |  |  |
| pMGMT-unmethylated | 39 | 1 |  |  |  | 39 | 1 |  |  |  |
| pMGMT-methylated | 48 | 0.27 (0.13-0.55) | **<0.001** |  |  | 48 | 0.206 (0.07-0.60) | **0.004** |  |  |
| Treatment data |  |  |  |  |  |  |  |  |  |  |
| Extent of resection (EOR) |  |  |  |  |  |  |  |  |  |  |
| Non-gross total resection(non-GTR) | 322 | 1 |  | 1 |  | 322 | 1 |  | 1 |  |
| Gross total resection (GTR) | 119 | 0.135 (0.08-0.22) | **<0.001** | 0.174 (0.07-0.41) | **<0.001** | 112 | 0.149 (0.08-0.28) | **<0.001** | 0.220 (0.05-1.02) | 0.052 |
| Postoperative ma-gement |  |  |  |  |  |  |  |  |  |  |
| Observation with MRI | 54 | 1 |  | 1 |  | 53 | 1 |  | 1 |  |
| Upfront chemotherapy alone | 55 | 0.672 (0.39-1.16) | 0.155 | 1 |  | 51 | 0.571 (0.29-1.11) | 0.10 | 1 |  |
| Radiotherapy or radio-chemotherapy | 299 | 0.596 (0.39-0.92) | **0.019** | 0.325 (0.15-1.69) | **0.003** | 287 | 0.87 (0.30-0.79) | **0.004** | 0.674 (0.48-0.95) | **0.024** |
| Disease progress |  |  |  |  |  |  |  |  |  |  |
| Yes | 193 |  |  |  |  | 164 |  |  |  |  |
| No | 250 |  |  |  |  | 385 |  |  |  |  |

Supplementary Table S4. Univariate and Multivariate Analysis of Prognostic Factors for PFS and OS of IDH-MT low-grade gliomas

|  | PFS | | | | | OS | | |
| --- | --- | --- | --- | --- | --- | --- | --- | --- |
|  |  | Univariate |  | Multivariable |  |  | Univariate |  |
|  | N | HR (CI 95%) | P value | HR (CI 95%) | P value | N | HR (CI 95%) | P value |
| No. of patients | 177 |  |  |  |  | 258 |  |  |
| Median age, y (IQR) | 43 (34-51) | 1.003 (0.98-1.03) | 0.849 |  |  | 43 (35-52) | 1.049 (1.01-1.09) | **0.007** |
| Gender |  |  |  |  |  |  |  |  |
| Male | 92 | 1 |  |  |  | 143 | 1 |  |
| Female | 85 | 1.532 (0.88-2.65) | 0.131 |  |  | 115 | 1.187 (0.58-2.43) | 0.639 |
| Tumor-associated epilepsy |  |  |  |  |  |  |  |  |
| No | 108 | 1 |  |  |  | 136 | 1 |  |
| Yes | 69 | 0.787 (0.45-1.36) | 0.391 |  |  | 122 | 0.63 (0.31-1.3) | 0.213 |
| Pathologic diagnosis |  |  |  |  |  |  |  |  |
| Oligodendroglioma  With Oligoastroglioma | 85 | 1 |  |  |  | 174 | 1 |  |
| Astrocytomas | 85 | 1.201 (0.70-2.05) | 0.503 |  |  | 82 | 0.97 (0.43-2.18) | 0.941 |
| Grade (2016 WHO CNS) |  |  |  |  |  |  |  |  |
| 2 | 119 | 1 |  | 1 |  | 133 | 1 |  |
| 3 | 58 | 2.417 (1.42-4.11) | **0.001** | 2.634 (1.26-5.51) | **0.010** | 125 | 1.845 (0.88-3.87) | 0.105 |
| Midline crossing |  |  |  |  |  |  |  |  |
| No | 132 | 1 |  | 1 |  | 124 | 1 |  |
| Yes | 45 | 3.619 (2.02-6.48) | **<0.001** | 1.488 (0.63-3.54) | 0.369 | 42 | 3.126 (0.95-10.429 | 0.061 |
| Tumor extension |  |  |  |  |  |  |  |  |
| 1 lobe | 74 | 1 |  | 1 |  | 70 |  |  |
| ≥ 2 lobes | 35 | 2.42 (1.0-5.85) | **0.049** | 4.604 (1.13-18.83) | **0.034** | 30 | 1.78 (0.36-8.91) | 0.482 |
| 1 or > 1 lobes with involvement of deep structures | 68 | 6.08 (2.85-13.0) | **<0.001** | 1.844 (0.39-8.75) | 0.441 | 66 | 2.90 (0.70-12.1) | 0.144 |
| Median pre-operative Tumoral Volume computed on T2-weighted images, cm3 (IQR) | 81.13 (41.95-147.34) |  |  |  |  | 80.38 (40.15-146.95) |  |  |
| >80 cm^3^ | 67 | 1 |  | 1 |  | 63 | NA | NA |
| ≤80 cm^3^ | 70 | 6.403 (2.25-18.24) | **<0.001** | 7.795 (0.78-10.06) | 0.116 | 64 | NA | NA |
| Molecular data |  |  |  |  |  |  |  |  |
| Ki-67 |  |  |  |  |  |  |  |  |
| Low expressed | 108 | 1 |  |  |  | 100 | 1 |  |
| High expressed | 63 | 1.689 (0.97-2.94) | 0.065 |  |  | 62 | 2.0 (0.57-6.98) | 0.277 |
| P53 |  |  |  |  |  |  |  |  |
| Low expressed | 95 | 1 |  |  |  | 154 |  |  |
| High expressed | 68 | 1.43 (0.81-2.53) | 0.218 |  |  | 95 | 1.841 (0.88-3.85) | 0.106 |
| ATRX |  |  |  |  |  |  |  |  |
| Positive | 104 | 1 |  |  |  | 155 | 1 |  |
| Negative | 52 | 1.626 (0.88-3.02) | 0.124 |  |  | 60 | 0.67 (0.19-2.32) | 0.528 |
| 1p/19q codeletion |  |  |  |  |  |  |  |  |
| 1p/19q-intact | 28 | 1 |  |  |  | 47 | 1 |  |
| 1p/19q-codeleted | 33 | 1.31 (0.37-4.63) | 0.675 |  |  | 106 | 0.645 (0.26-1.59) | 0.349 |
| pMGMT methylation |  |  |  |  |  |  |  |  |
| pMGMT-unmethylated | 7 | 1 |  |  |  | 7 | NA | NA |
| pMGMT-methylated | 33 | 0.359 (0.06-2.06) | 0.25 |  |  | 33 | NA | NA |
| pTERT mutation |  |  |  |  |  |  |  |  |
| pTERT-intact | 10 | 1 |  |  |  | 20 | 1 |  |
| pTERT-mutant | 7 | 0.817 (0.05-13.24) | 0.887 |  |  | 51 | 1.994 (0.24-16.29) | 0.519 |
| Treatment data |  |  |  |  |  |  |  |  |
| Extent of resection (EOR) |  |  |  |  |  |  |  |  |
| Non-gross total resection(non-GTR) | 101 | 1 |  |  |  | 99 | 1 |  |
| Gross total resection (GTR) | 76 | 0.175 (0.09-0.36) | **<0.001** | 0.273 (0.09-0.87) | **0.029** | 67 | 0.274 (0.07-1.05) | 0.058 |
| Postoperative management |  |  |  |  |  |  |  |  |
| Observation with MRI | 14 | 1 |  |  |  | 13 | NA | NA |
| Upfront chemotherapy alone | 25 | 0.544 (0.19-1.52) | 0.245 |  |  | 22 | NA | NA |
| Radiotherapy or radio-chemotherapy | 131 | 0.564 (0.24-1.36) | 0.200 |  |  | 124 | NA | NA |
| Events |  |  |  |  |  |  |  |  |
| Yes | 56 |  |  |  |  | 30 |  |  |
| No | 121 |  |  |  |  | 227 |  |  |

Supplementary Table S5. Univariate and Multivariate Analysis of Prognostic Factors for PFS and OS of IDH-WT low-grade gliomas

|  | PFS | | | | | OS | | | | |
| --- | --- | --- | --- | --- | --- | --- | --- | --- | --- | --- |
|  |  | Univariate |  | Multivariable |  |  | Univariate |  | Multivariable |  |
|  | N | HR (CI 95%) | P value | HR (CI 95%) | P value | N | HR (CI 95%) | P value | HR (CI 95%) | P value |
| No. of patients | 73 |  |  |  |  | 88 |  |  |  |  |
| Median age, y (IQR) | 52 (42-61) | 1.038 (1.00-1.07) | **0.012** | 1.036 (0.99-1.08) | 0.102 | 52 (39.75-61) | 1.061 (1.03-1.09) | **<0.001** | 1.079 (1.02-1.14) | **0.006** |
| Gender |  |  |  |  |  |  |  |  |  |  |
| Male | 41 | 1 |  |  |  | 48 | 1 |  |  |  |
| Female | 32 | 0.641 (0.32-1.40) | 0.238 |  |  | 40 | 0.661 (0.33-1.31) | 0.270 |  |  |
| Tumor-associated epilepsy |  |  |  |  |  |  |  |  |  |  |
| No | 54 | 1 |  |  |  | 56 | 1 |  |  |  |
| Yes | 19 | 0.316 (0.12-0.83) | **0.020** | 1.02 (0.32-3.26) | 0.973 | 32 | 0.652 (0.33-1.30) | 0.226 |  |  |
| Pathologic diagnosis |  |  |  |  |  |  |  |  |  |  |
| Oligodendroglioma  With Oligoastroglioma | 12 | 1 |  |  |  | 29 | 1 |  |  |  |
| Astrocytomas | 60 | 0.882 (0.35-2.15) | 0.786 |  |  | 59 | 0.647 (0.33-1.26) | 0.199 |  |  |
| Grade (2016 WHO CNS) |  |  |  |  |  |  |  |  |  |  |
| 2 | 33 | 1 |  | 1 |  | 40 | 1 |  | 1 |  |
| 3 | 40 | 4.037 (1.74-9.37) | **0.001** | 3.06 (0.97-9.70) | 0.057 | 48 | 4.977 (2.16-11.48) | **< 0.001** | 4.624 (0.95-22.46) | 0.057 |
| Midline crossing |  |  |  |  |  |  |  |  |  |  |
| No | 57 | 1 |  |  |  | 55 | 1 |  |  |  |
| Yes | 16 | 1.403 (0.63-3.13) | 0.409 |  |  | 16 | 1.066 (0.40-2.87) | 0.899 |  |  |
| Tumor extension |  |  |  |  |  |  |  |  |  |  |
| 1 lobe | 20 | 1 |  | 1 |  | 19 | 1 |  | 1 |  |
| ≥ 2 lobes | 14 | 7.43 (1.46-37.8) | **0.016** | 11.483 (1.26-104.53) | **0.03** | 14 | 3.40 (0.66-17.5) | 0.143 | 1 |  |
| 1 or > 1 lobes with involvement of deep structures | 39 | 11.8 (2.77-50.5) | **< 0.001** | 20.412 (2.15-193.41) | **0.009** | 38 | 6.75 (1.54-29.5) | **0.011** | 1.736 (1.08-2.77) | **0.020** |
| Median pre-operative Tumoral Volume computed on T2-weighted images, cm^3^ (IQR) | 61.73 (29.17-130.21) |  |  |  |  | 59.55 (28.73-127.66) |  |  |  |  |
| >60 cm^3^ | 28 | 1 |  |  |  | 28 | 1 |  |  |  |
| ≤60 cm^3^ | 39 | 2.19 (0.91-5.30) | 0.081 |  |  | 27 | 2.01 （0.78-5.19） | 0.150 |  |  |
| Molecular data |  |  |  |  |  |  |  |  |  |  |
| Ki-67 |  |  |  |  |  |  |  |  |  |  |
| Low expressed | 34 | 1 |  | 1 |  | 34 | 1 |  |  |  |
| High expressed | 37 | 4.5 (1.96-10.32) | **< 0.001** | 0.591 (0.16-2.12) | 0.418 | 36 | 7.201 (2.42-21.4) | **< 0.001** | 0.851 (0.18-3.88) | 0.834 |
| P53 |  |  |  |  |  |  |  |  |  |  |
| Low expressed | 47 | 1 |  |  |  | 59 | 1 |  |  |  |
| High expressed | 22 | 1.3 (0.62-2.71) | 0.484 |  |  | 26 | 1.397 (0.70-2.78) | 0.341 |  |  |
| ATRX |  |  |  |  |  |  |  |  |  |  |
| Positive | 61 | 1 |  |  |  | 65 | 1 |  |  |  |
| Negative | 5 | 0.287 (0.04-2.13) | 0.222 |  |  | 7 | 0.987 (0.30-2.84) | 0.982 |  |  |
| pMGMT methylation |  |  |  |  |  |  |  |  |  |  |
| pMGMT-unmethylated | 10 | 1 |  |  |  | 10 | 1 |  |  |  |
| pMGMT-methylated | 5 | 1.847 (0.31-11.19) | 0.504 |  |  | 5 | 0.893 (0.17-4.66) | 0.893 |  |  |
| pTERT mutation |  |  |  |  |  |  |  |  |  |  |
| pTERT-intact | 3 | 1 |  |  |  | 8 | 1 |  |  |  |
| pTERT-mutant | 6 | 1.447 (0.14-14.81) | 0.756 |  |  | 6 | 2.166 (0.46-10.3) | 0.331 |  |  |
| Treatment data |  |  |  |  |  |  |  |  |  |  |
| Extent of resection (EOR) |  |  |  |  |  |  |  |  |  |  |
| Non-gross total resection(non-GTR) | 59 | 1 |  |  |  | 60 | NA | NA |  |  |
| Gross total resection (GTR) | 16 | 0.060 (0.01-0.45) | **0.006** |  |  | 13 | NA | NA |  |  |
| Postoperative management |  |  |  |  |  |  |  |  |  |  |
| Observation with MRI | 9 | 1 |  |  |  | 9 | 1 |  |  |  |
| Upfront chemotherapy alone | 9 | 1.38 (0.36-5.33) | 0.639 |  |  | 9 | 1.40.(0.28-6.96) | 0.683 |  |  |
| Radiotherapy or radio-chemotherapy | 48 | 0.796 (0.27-2.38) | 0.683 |  |  | 46 | 1.12 (0.33-3.89) | 0.854 |  |  |
| Events |  |  |  |  |  |  |  |  |  |  |
| Yes | 33 |  |  |  |  | 35 |  |  |  |  |
| No | 42 |  |  |  |  | 55 |  |  |  |  |

Supplementary Table S6. Univariate and Multivariate Analysis of Prognostic Factors for PFS and OS of High-grade gliomas

|  | PFS | | | | | OS | | | | |
| --- | --- | --- | --- | --- | --- | --- | --- | --- | --- | --- |
|  |  | Univariate |  | Multivariable |  |  | Univariate |  | Multivariable |  |
|  | N | HR (CI 95%) | P value | HR (CI 95%) | P value | N | HR (CI 95%) | P value | HR (CI 95%) | P value |
| No. of patients | 143 |  |  |  |  | 154 |  |  |  |  |
| Median age, y (IQR) | 52 (44-59) | 1.012 (0.99-1.03) | 0.241 |  |  | 52 (44-59) | 1.01 (0.99-1.03) | 0.449 |  |  |
| Gender |  |  |  |  |  |  |  |  |  |  |
| Male | 91 | 1 |  |  |  | 96 | 1 |  |  |  |
| Female | 52 | 0.867 (0.55-1.36) | 0.535 |  |  | 58 | 0.775 (0.50-1.21) | 0.260 |  |  |
| Tumor-associated epilepsy |  |  |  |  |  |  |  |  |  |  |
| No | 137 | 1 |  |  |  | 130 | 1 |  |  |  |
| Yes | 16 | 0.785 (0.39-1.57) | 0.494 |  |  | 24 | 0.653 (0.37-1.15) | 0.139 |  |  |
| Pathologic diagnosis |  |  |  |  |  |  |  |  |  |  |
| Astrocytomas | 9 | 1 |  |  |  | 8 | 1 |  |  |  |
| Glioblastoma | 131 | 0.775 (0.31-1.92) | 0.583 |  |  | 146 | 0.722 (0.26-1.99) | 0.530 |  |  |
| Midline crossing |  |  |  |  |  |  |  |  |  |  |
| No | 94 | 1 |  |  |  | 92 | 1 |  | 1 |  |
| Yes | 49 | 1.565 (0.97-2.48) | 0.056 |  |  | 47 | 1.698 (1.06-2.73) | **0.029** | 1.621 (1.94-2.79) | 0.081 |
| Tumor extension |  |  |  |  |  |  |  |  |  |  |
| 1 lobe | 19 | 1 |  |  |  | 19 | 1 |  |  |  |
| ≥ 2 lobes | 27 | 1.60 (0.72-3.56) | 0.247 |  |  | 26 | 1.69 (0.72-4.0) | 0.231 |  |  |
| 1 or > 1 lobes with involvement of deep structures | 97 | 1.57 (0.78-3.18) | 0.208 |  |  | 94 | 1.51 (0.72-3.18) | 0.275 |  |  |
| Median pre-operative Tumoral Volume computed on T2-weighted images, cm^3^ (IQR) | 144.23 (102.57-172.67) |  |  |  |  | 141.32 (101.83-173.58) |  |  |  |  |
| >140 cm^3^ | 46 | 1 |  |  |  | 46 | 1 |  |  |  |
| ≤140 cm^3^ | 49 | 0.853 (0.50-1.47) | 0.568 |  |  | 47 | 0.703 (0.41-1.12) | 0.205 |  |  |
| Molecular data |  |  |  |  |  |  |  |  |  |  |
| Ki-67 |  |  |  |  |  |  |  |  |  |  |
| Low expressed | 3 | NA | NA |  |  | 3 | NA | NA |  |  |
| High expressed | 118 | NA | NA |  |  | 117 | NA | NA |  |  |
| P53 |  |  |  |  |  |  |  |  |  |  |
| Low expressed | 38 | 1 |  |  |  | 44 | 1 |  |  |  |
| High expressed | 67 | 0.734 (0.44-1.24) | 0.246 |  |  | 75 | 1.453 (0.83-2.55) | 0.192 |  |  |
| ATRX |  |  |  |  |  |  |  |  |  |  |
| Positive | 89 | 1 |  |  |  | 96 | 1 |  |  |  |
| Negative | 10 | 0.511 (0.20-1.28) | 0.152 |  |  | 16 | 0.829 (0.37-1.84) | 0.647 |  |  |
| IDH |  |  |  |  |  |  |  |  |  |  |
| Wild type | 125 | 1 |  | 1 |  | 132 | 1 |  | 1 |  |
| Mutation | 10 | 0.339 (0.14-0.84) | **0.021** | 0.324 (0.12-0.86) | **0.023** | 14 | 0.284 (0.11-0.71) | **0.007** | 0.432 (0.15-1.23) | 0.115 |
| pMGMT methylation |  |  |  |  |  |  |  |  |  |  |
| pMGMT-unmethylated | 22 | 1 |  |  |  | 22 | 1 |  |  |  |
| pMGMT-methylated | 10 | 0.329 (0.11-1.02) | 0.054 |  |  | 10 | 0.161 (0.02-1.35) | 0.092 |  |  |
| pTERT mutation |  |  |  |  |  |  |  |  |  |  |
| pTERT-intact | 8 | 1 |  |  |  | 8 | 1 |  |  |  |
| pTERT-mutant | 10 | 2.263 (0.60-8.60) | 0.23 |  |  | 11 | 1.049 (0.09-11.71) | 0.970 |  |  |
| EGFR amplification |  |  |  |  |  |  |  |  |  |  |
| EGFR-intact | 5 | 1 |  |  |  | 5 | NA | NA |  |  |
| EGFR-amplified | 2 | 4.472 (0.28-71.8) | 0.290 |  |  | 2 | NA | NA |  |  |
| Treatment data |  |  |  |  |  |  |  |  |  |  |
| Extent of resection (EOR) |  |  |  |  |  |  |  |  |  |  |
| Non-gross total resection(non-GTR) | 127 | 1 |  | 1 |  | 124 | 1 |  | 1 |  |
| Gross total resection (GTR) | 16 | 0.297 (0.13-0.69) | **0.005** | 0.297 (0.12-0.72) | **0.007** | 15 | 0.416 (0.19-0.91) | **0.028** | 0.545 (0.23-1.29) | 0.169 |
| Postoperative management |  |  |  |  |  |  |  |  |  |  |
| Observation with MRI | 23 | 1 |  | 1 |  | 22 | 1 |  | 1 |  |
| Upfront chemotherapy alone | 11 | 0.907 (0.32-2.58) | 0.856 | 1 |  | 11 | 0.681 (0.27-1.71) | 0.413 | 1 |  |
| Radiotherapy or radio-chemotherapy | 93 | 0.380 (0.19-0.78) | **0.008** | 0.671 (0.49-0.91) | **0.011** | 90 | 0.191 (0.10-0.36) | **<0.001** | 0.475 (0.35-0.64) | **<0.001** |
| Events |  |  |  |  |  |  |  |  |  |  |
| Yes | 86 |  |  |  |  | 89 |  |  |  |  |
| No | 57 |  |  |  |  | 65 |  |  |  |  |

Supplementary Table S7. Multivariate Analysis of Prognostic Factors for OS of High-grade gliomas.

| Variables | HR (CI 95%) | P value |
| --- | --- | --- |
| IDH | 0.433 (0.153-1.226) | 0.115 |
| Midline crossing | 1.735 (1.002-3.006) | **0.049** |
| Gross total resection (GTR) | 0.616 (0.257-1.477) | 0.278 |
| Radiotherapy or radio-chemotherapy | 0.437 (0.316-0.603) | **<0.001** |
